# Are we missing the Institute of Medicine’s mark? A systematic review of patient-reported outcome measures assessing quality of patient-centred cancer care

**DOI:** 10.1186/1471-2407-14-41

**Published:** 2014-01-25

**Authors:** Flora Tzelepis, Shiho K Rose, Robert W Sanson-Fisher, Tara Clinton-McHarg, Mariko L Carey, Christine L Paul

**Affiliations:** 1 Health Behaviour Research Group, Priority Research Centre for Health Behaviour, University of Newcastle & Hunter Medical Research Institute, Newcastle, New South Wales, Australia

**Keywords:** Patient-centred, Quality of care, Patient-reported outcome measures, Cancer, Reliability, Validity, Systematic review

## Abstract

**Background:**

The Institute of Medicine (IOM) has endorsed six dimensions of patient-centredness as crucial to providing quality healthcare. These dimensions outline that care must be: 1) respectful to patients’ values, preferences, and expressed needs; 2) coordinated and integrated; 3) provide information, communication, and education; 4) ensure physical comfort; 5) provide emotional support—relieving fear and anxiety; and 6) involve family and friends. However, whether patient-reported outcome measures (PROMs) comprehensively cover these dimensions remains unexplored. This systematic review examined whether PROMs designed to assess the quality of patient-centred cancer care addressed all six IOM dimensions of patient-centred care and the psychometric properties of these measures.

**Methods:**

Medline, PsycINFO, Current Contents, Embase, CINAHL and Scopus were searched to retrieve published studies describing the development and psychometric properties of PROMs assessing the quality of patient-centred cancer care. Two authors determined if eligible PROMs included the six IOM dimensions of patient-centred care and evaluated the adequacy of psychometric properties based on recommended criteria for internal consistency, test-retest reliability, face/content validity, construct validity and cross-cultural adaptation.

**Results:**

Across all 21 PROMs, the most commonly included IOM dimension of patient-centred care was “information, communication and education” (19 measures). In contrast, only five measures assessed the “involvement of family and friends.” Two measures included one IOM-endorsed patient-centred care dimension, two measures had two dimensions, seven measures had three dimensions, five measures had four dimensions, and four measures had five dimensions. One measure, the Indicators (Non-small Cell Lung Cancer), covered all six IOM dimensions of patient-centred care, but had adequate face/content validity only. Eighteen measures met the recommended adequacy criteria for construct validity, 15 for face/content validity, seven for internal consistency, three for cross-cultural adaptation and no measure for test-retest reliability.

**Conclusions:**

There are no psychometrically rigorous PROMs developed with cancer patients that capture all six IOM dimensions of patient-centred care. Using more than one measure or expanding existing measures to cover all six patient-centred care dimensions could improve assessment and delivery of patient-centred care. Construction of new comprehensive measures with acceptable psychometric properties that can be used with the general cancer population may also be warranted.

## Background

The Institute of Medicine has defined high quality health care as the provision of appropriate services in a technically competent manner, and includes good communication, shared decision-making and is consistent with patient values and preferences [1]. Optimizing the *structure* (e.g., hospital resources, number of staff), *processes* (e.g., interactions between health care providers and patients, use of effective therapies) and *outcomes* (e.g., survival, quality of life) of health care services are crucial to achieving high quality care [1]. In 2001, the IOM published “Crossing the Quality Chasm” a broad framework which recommended improvements to the following six areas of healthcare in order to achieve high quality care: safety; effectiveness; timeliness; efficiency; equity; and patient-centredness [1]. Within the area of patient-centredness, the IOM also endorsed Gerteis et al’s six dimensions of patient-centred care [2] which state that care must be: 1) respectful to patients’ values, preferences, and expressed needs; 2) coordinated and integrated; 3) provide information, communication, and education; 4) ensure physical comfort; 5) provide emotional support—relieving fear and anxiety; and 6) involve family and friends [1]. The IOM’s recognition of patient-centredness as an indicator of quality acknowledges the adoption of a *whole-person* orientation to healthcare that goes beyond solely focusing on treatment of the disease.

A variety of sources have been used to assess the quality of care that patients receive including administrative databases, cancer registries, medical records, patient self-reported measures, physician surveys, and pharmacy and laboratory data [3]. However unlike other aspects of quality, such as efficiency, patient self-report is arguably the only way to assess constructs that relate to patient-centredness. For instance, the severity of cancer pain and levels of fatigue experienced by a patient can only reliably be assessed by the patient themselves, and self-report is widely recognised as the gold standard for such assessments [4]. The value of obtaining patient self-report data is further demonstrated by research reporting that patients’ perceptions of quality of health care have been associated with important medical and psychological outcomes, including quality of life [5-8], anxiety and depression [6-9]. Patients’ perceptions of quality of care have also been associated with factors that directly affect the effectiveness and efficiency of health care such as the under-utilisation of treatments [10-12] and mistrust of the medical system [13,14].

Patient-reported outcome measures (PROMs) that have been designed to assess the quality of patient-centred care include measures of: 1) *satisfaction with care*; and 2) *experiences of care*. Satisfaction with care measures investigate the extent to which an individual’s health care experiences met his/her expectations [15]. However, a range of factors unrelated to the actual health care that was delivered, such as differences among patients’ expectation levels, can cause variability in satisfaction ratings, which reduce their reliability for widespread and ongoing monitoring of attempts to improve patient-centred care [15]. In contrast, experiences of care measures ask patients to indicate what actually happened during the process of care delivery, and so are less influenced by subjective patient expectations and provide more detailed information to health care providers and systems about where quality improvements are needed [16,17]. However, in order to accurately reflect the quality of care received and identify variations in patients’ experiences, PROMs should meet recommended psychometric criteria for reliability (internal consistency, test re-test reliability), and validity (face, content, construct validity) [18-24].

There are few existing reviews that have assessed the psychometric properties of measures developed to identify patients’ experiences of care across a range of settings and diseases [25-28]. Only one of these reviews evaluated the psychometric properties of quality of care measures designed specifically for cancer patients, but focused on satisfaction measures [27]. Further, this review [27] did not investigate the degree to which these quality of care measures assessed the six IOM-endorsed dimensions of patient-centred care [1].

This systematic review identified:

1) the degree to which PROMs developed to assess the quality of patient-centred cancer care since the publication of the IOM’s “Crossing the Quality Chasm” report in 2001 have addressed the IOM’s six endorsed dimensions of patient-centred care [1]; and

2) the psychometric properties of these measures.

## Methods

### Search strategy and selection criteria

The electronic databases Medline, PsycINFO, Current Contents, Embase, CINAHL and Scopus were searched to retrieve published studies outlining the development of PROMs designed to assess the quality of patient-centred cancer care. Given the IOM’s *Crossing the Quality Chasm* report was published in 2001 [1], databases were searched between January 2001 and December 2011 inclusive. The following combinations of keywords were used: (patient-centred or patient-centered or quality of care or satisfaction or experience*) AND (questionnaire* or survey* or instrument* or measure* or scale* or tool*) AND (cancer* or neoplasm* or oncol*). The use of an * in the keywords allows words that contain that term to be captured in the literature search. For example the keyword measure* will identify articles that contain variations of that word such as measure, measures, measurement and measurements. The reference lists of retrieved articles were also checked to identify any additional relevant publications.

The inclusion criteria for this systematic review were studies that:

(i) reported the development and psychometric properties (reliability and validity) of new PROMs designed to assess the quality of patient-centred cancer care, or reported the validation of an existing measure for use with a new population (e.g. patient-centred care measure translated for use with a Spanish cancer patient population). Given the IOM’s recommendations were published in 2001 [1], studies describing the validation of an existing measure were eligible only if the original PROM was developed from 2001 onwards.

(ii) described PROMs specifically developed for use with adult cancer patient populations (i.e., aged 18 years or older); and

(iii) were published in an English language peer-reviewed journal.

Publications were excluded if they:

(i) were reviews, editorials, commentaries or protocol papers;

(ii) reported qualitative research or used a Delphi consensus process;

(iii) reported data from medical records, administrative databases or cancer registries (i.e., patients were not surveyed);

(iv) focussed on cancer screening only;

(v) predominately surveyed cancer patients under 18 years of age;

(vi) assessed the views of health professionals such as oncologists, nurses, and general practitioners;

(vii) examined the perceptions of relatives and/or caregivers;

(viii) included only cancer patients with advanced cancer or those receiving end of life care; These patients were excluded because the outcome measures and care delivered to patients with advanced cancer can be unique, reflecting the specific goals of advanced disease and/or end-of-life care [29].

(ix) reported only patient ratings of quality of care and/or patient characteristics associated with quality of care – i.e. did not develop a measure with the aim of testing its psychometric properties; and

(x) validation of an existing measure that was not eligible for the review (e.g. the original PROM was developed prior to 2001). PROMs developed prior to 2001 were excluded because it would have been unreasonable to assess the degree to which such PROMs addressed the IOM’s dimensions of patient-centred care given the IOM recommendations were published in 2001 [1].

### Study and sample characteristics

The study and sample characteristics extracted from eligible publications included: the name of the measure; country of development; patient recruitment setting (e.g. hospital, cancer registry); patient eligibility criteria; sample size; consent rate; participants’ socio-demographic characteristics (e.g. mean age, gender, level of education, employment status); and participants’ disease and treatment characteristics (e.g. cancer type, cancer stage and/or time since diagnosis, treatments received).

### Items and subscales of measures

Information extracted about the characteristics of each measure included: the type of measure (i.e. satisfaction versus experiences); number of items; the type of response scale, and the names and number of subscales. Two coders (FT & SKR) independently examined each of the PROMs’ items to determine whether or not the PROM contained content that related to any of the IOM’s six patient-centred dimensions and how many of the six IOM-endorsed dimensions of patient-centred care were covered [1]. At least one item in the PROM needed to examine issues related to a particular IOM patient-centred care dimension (as defined below) for that area to be categorised as addressed. A conservative approach was taken when deciding whether or not a measure covered a particular dimension. For example, if a measure included an item that examined whether a patient *was provided with information on long-term side effects,* the measure was categorised as meeting the information and communication dimension, but not the physical comfort dimension. The physical comfort dimension was classified as present only if items assessed the provision of pain relief or the management of physical symptoms. The criteria used to classify each patient-centred care dimension, which are based on the definitions outlined in the IOM’s “Crossing the Quality Chasm” report [1], are described below. Only one aspect of the dimension was needed for the PROM to be classed as covering that patient-centred care dimension.

#### 1) Respect for patients’ values, preferences, and expressed needs

PROMs were classified as covering this dimension if they assessed: a) whether care responded to the patient’s cultural and other values, preferences and needs; b) whether patients were given the opportunity to express their views; c) whether patients were treated with respect during care; and/or d) whether patients were informed and involved in decision making according to their preferences [1].

#### 2) Coordinated and integrated care

PROMs were rated as containing this dimension if they asked: a) whether patient care was coordinated and integrated; b) whether there was timely transfer of up-to-date patient information between healthcare professionals; and/or c) whether patient transitions from one healthcare setting to another went smoothly [1].

#### 3) Provide information, communication, and education

PROMs met the criteria for this dimension if they examined whether health care professionals: a) communicated with patients in a way they could understand; and/or b) provided accurate information regarding care including diagnosis, prognosis, treatment options, follow-up care and support services, according to the patient’s preferred level of information provision [1].

#### 4) Physical comfort

PROMs were classified as covering this dimension if they asked patients whether health care professionals: a) promptly provided pain relief; and/or b) attended to the patient’s physical symptoms and needs [1].

#### 5) Emotional support

PROMs were categorised as meeting this dimension if they assessed whether healthcare professionals: a) addressed the patients’ emotional and spiritual concerns, such as anxiety, which could be experienced for a variety of reasons including uncertainty about their disease, concerns about the financial impact of treatment, or worrying about the impact of the illness on their family [1].

#### 6) Involvement of family and friends

PROMs were considered to have met this dimension if they assessed whether: a) family and friends were involved in the patient’s decision making and care according to the patient’s preferences; and/or b) whether care was responsive to the concerns of family and friends and recognised their needs [1].

Two coders (FT & SKR) also independently examined which PROMs covered *all* aspects within each of the IOM dimensions. For instance in terms of the physical comfort dimension, PROMs that included items that addressed both of the following criteria were identified: a) promptly provided pain relief; *and* b) attended to the patient’s physical symptoms and needs.

### Psychometric properties of measures

The psychometric properties of each measure were assessed against the same criteria used by Clinton-McHarg and colleagues in their review of instruments designed to measure the psychosocial health of adolescent and young adult cancer survivors [30]. The psychometric criteria are described below.

#### Internal consistency

A measure was coded as having acceptable internal consistency if correlations for the total scale and each subscale were calculated [19] and a Cronbach’s alpha >0.70 (continuous or dichotomous scales) or Kuder-Richardson 20 (KR-20) >0.70 was reported for the total scale and each sub-scale [18,19].

#### Test-retest reliability

Measures were recorded as having adequate test-retest reliability if the instrument had been administered twice to the same sample and: 1) the second administration occurred within 2-14 days of the first administration [20]; and 2) correlations for the total scale, subscales and items were calculated [21] and the agreement between scores achieved a Cohen’s kappa co-efficient (κ) > 0.60 (nominal or ordinal scales) [19] or Pearson correlation coefficient (r) > 0.70 (interval scales) [18,19] or intraclass correlation coefficient (ICC) >0.70 (interval scales) [18,19].

#### Face validity

Measures were considered to have face validity if both those who administered it, and those who completed it, agreed it appeared to measure what it was designed to measure [22].

#### Content validity

A measure was reported to have adequate content validity if the following processes were described: 1) how the items were developed or selected [18,19]; 2) how and by whom the content was assessed [18,19]; and 3) if modifications to the content were needed that the revisions addressed the issues identified [18,19].

#### Construct validity

Each measure was assessed as having adequate construct validity if any of the following tests were performed: 1) comparison with other existing measures [19] resulting in Pearson correlation coefficients of (r) >0.40 (convergent validity) or (r) < 0.30 (divergent validity) [23]; 2) comparison of scores on the measure differ significantly between groups with known differences (discriminative validity) [18]; or 3) factor analysis [19] with Eigenvalues set at > 1 [24].

#### Cross-cultural adaptation

A measure was considered to have adequate cross-cultural adaptation if a conceptually and linguistically equivalent version of the original form confirmed the reliability and validity reflected in the original measure [18].

### Coding process

Two authors (FT & SKR) independently assessed all potentially relevant publications to determine whether they met eligibility for inclusion in the review. There was 84% agreement between the two coders’ ratings. Where discrepancies emerged, inconsistent ratings were discussed between the coders until consensus was reached. Both coders also independently extracted information for the Tables from included publications to ensure accuracy. The coders then compared the information extracted and discussed any inconsistencies until agreement was reached.

## Results

### Study eligibility

**Figure 1:**
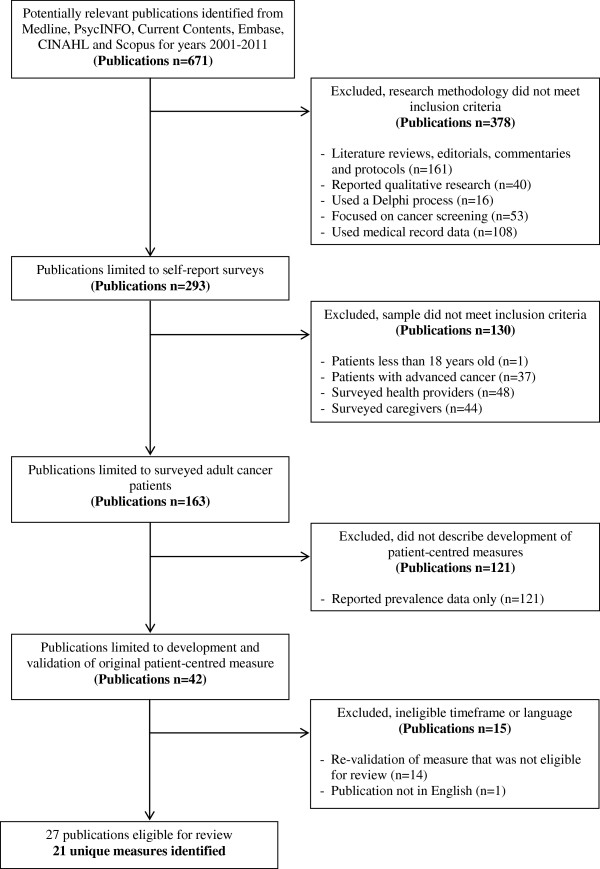
Flowchart of methods used to identify relevant publications.

A total of 671 publications were identified from the electronic database searches and publication reference lists. Of these, 161 publications were reviews, editorials, commentaries or protocol papers, 40 reported qualitative research and 16 used a Delphi consensus process and were excluded. A further 108 papers reported data from medical records, administrative databases or cancer registries and 53 focussed on cancer screening only and were removed. Of the 293 remaining publications, 48 assessed the views of health professionals such as oncologists, nurses, and general practitioners, 44 focussed on the perceptions of relatives or caregivers, one related to cancer patients aged under 18 years, and 37 focused on an advanced cancer population and/or those receiving end-of-life care and were excluded. Of the remaining 163 publications that surveyed adult cancer patients, 121 examined the prevalence of features of care and/or characteristics associated with patient experiences and 14 validated an existing measure that was not eligible for the review (e.g. the original PROM was developed prior to 2001). One paper that reported the development of the EORTC OUT-PATSAT35 was published in French and therefore excluded [31]. This left 27 papers that reported the development of an instrument and its psychometric properties with an adult cancer patient population, or reported the psychometric properties of a re-validated measure for use with a new population. In these papers, 21 unique PROMs were described (see Figure 1).

### Setting and Sample Characteristics

Table 1 provides a detailed description of the setting and sample characteristics of the eligible studies [32-55]. Six studies were conducted in the USA [32,35,42-44,47], five in The Netherlands [37,39,40,50,52], three in England [41,49,54], two in France [53,55], and one in Australia [33], Canada [34], Europe and Asia [38], Germany [46] and Japan [48]. Seventeen studies recruited cancer patients from hospitals or treatment centres [33,34,38-44,46-50,53-55], whereas only one study recruited patients via a population-based cancer registry [32]. The sample sizes in each study ranged from 82 to 2659 cancer patients and the consent rates varied from 43% to 85%. Thirteen studies included more than one cancer type [32-35,38,41,43,44,46-48,52,55].

**Table 1:** Sample characteristics of studies that have developed PROMs assessing quality of patient-centred cancer care

**Measure**	**Sample size**	**Consent rate**	**Eligibility criteria**	**Setting & country**	**Socio-demographics**	**Cancer type, stage/ diagnosis**	**Cancer treatments**
Assessment of Patient Experiences of Cancer Care (APECC) [32]	623	69.2% participation rate.	Read English, diagnosed with leukaemia or bladder or colorectal cancer between June 1999-May 2001 (i.e., 2-5 years before study enrolment), at least 20 years old at diagnosis, have received cancer treatment, have the cancer of interest as their first cancer diagnosis, not have any other cancer between their initial diagnosis and the start of the study, have no objections from their physician of record to their participation.	Cancer Prevention Institute of California’s cancer registry, USA.	43.3% women	Colorectal cancer: 59.6%	38.7% surgery only
		49.2% response rate.			37.7% aged 50-64 years	Bladder cancer: 26.2%	35.1% surgery plus chemotherapy or radiation
					20.3% college degree	Leukaemia: 14.3%	
					71.5% married/defacto		
					73.8% Non-Hispanic white	84.4% in remission.	12.3% surgery plus chemotherapy and radiation
					83.6% private health insurance	Mean of 3.5 years since diagnosis.	13.9% chemotherapy with or without radiation but no surgery
Cancer Care Coordination Questionnaire for Patients [33]	686	-	Sample 1 (n = 245): Patients were in follow-up for any cancer that had been treated between 3 to 12 months previously, had sufficient English and were not cognitively impaired and were not receiving end of life care.	Sample 1: Six centres (2 metropolitan & 4 regional).	Total participants:	Colorectal: 82.5%	96% surgery
					46.8% women	Gynaecological: 7.6%	40.5% chemotherapy
					Mean age: 66.1 years	Breast: 2.6%	12.2% radiotherapy
					66.9% married/defacto	Lung/mesothelioma: 1.3%	3.9% hormone therapy
				Sample 2: 22 public and private hospitals in metropolitan and regional centres.	35.3% tertiary degree or diploma	Other/multiple sites: 4.7%	
					23.5% employed full-time	Primary cancer: 91.8%	
						Recurrent cancer: 3.8%	
			Sample 2 (n = 441): Patients with a newly diagnosed colorectal cancer undergoing initial surgical treatment.	Australia			
Cancer Patient Information Importance and Satisfaction Tool [34]	540	-	-	Ambulatory setting of regional cancer centre, Canada.	53% women	Breast: 19.1%	-
					Mean age: 60.9 years	Haematological: 12.5%	
						Genitourinary: 12.5%	
						Skin: 11.5%	
						Gastrointestinal: 11.5%	
						Head and neck: 11.3%	
						Gynecologic: 11.2%	
						Lung: 10.4%	
						21.3% diagnosed in last year, 48.3% between 2-5 years ago.	
Cancer Therapy Satisfaction Questionnaire (CTSQ) [35,36]	361	-	Provided written informed consent, aged 18 years or older, read and write in English, available for follow-up evaluation, actively receiving more than one cycle of first- or second-line chemo, biological or hormonal therapy for early or advanced cancer, mentally and physically capable of participation.	14 community clinical practices, USA.	63.2% women	Breast: 37.9%	First-line of therapy: 48.8%
					Mean age: 60.7 years	Colorectal: 33.5%	
					90.9% white	Lung: 25.2%	Second-line of therapy: 28.0%
					16.3% college/university degree	Melanoma: 3.3%	
						Stage I: 6.1%	Adjuvant: 23.3%
						Stage II: 25.2%	
						Stage III: 24.9%	
						Stage IV: 43.8%	
Consumer Quality Index Breast Care (CQI-BC) [37]	731	63%	Older than 18 years, having received breast care in the last 24 months, not being approached in the past for CQI surveys.	Selected from claims data of four health insurance companies, The Netherlands.	99.7% women	Breast cancer: 57%	-
					30% aged between 55 and 64 years	Benign breast disorder: 38%	
						Breast carcinoma in situ: 5%	
						-	
EORTC cancer in-patient satisfaction with care measure (EORTC IN-PATSAT32) [38]	647	84.9%	Diagnosed with cancer, aged 18 years or older, hospitalized for at least three days, mentally able to complete questionnaire.	Surgery or medical oncology wards in hospitals, Belgium, France, Germany, Italy, Poland, Spain, Sweden, Taiwan, United Kingdom.	59% women	Breast: 35.1%	Current or planned treatment:
					Median age: 57 years	Gastro-intestinal: 17.2%	55% surgery
					21% university educated	Gynaecologic: 10%	
					73.6% married/defacto	Head and neck: 7.3%	40.8% chemotherapy
					40.5% full time employed	Genito-urinary: 7.1%	3.2% surgery and chemotherapy
						Haematological: 6.5%	
						Respiratory: 5.6%	0.9% other
						Bone: 2.8%	
						Brain: 1.5%	
						Melanoma: 0.8%	
						Other: 6.2%	
						73.9% local/loco-regional	
						26% metastatic	
						Median of 15 weeks since diagnosis.	
Indicators (Head & Neck Cancer) [39]	158	84%	Patients with head and neck cancer newly diagnosed between May to December 2003	Selected from clinic lists at a university hospital, The Netherlands	27% women	Head & neck cancer.	First treatment:
					Mean age: 62 years	Larynx and hypharynx: 38%	Operation: 56%
					23% highly educated	Cavity of the mouth: 36%	Radiotherapy: 37%
						Other: 26%	Chemotherapy: 7%
						-	
Indicators (Non-small Cell Lung Cancer) [40]	100	76%	Patients newly diagnosed with non-small cell lung cancer between September 2004 and February 2005.	6 hospitals, The Netherlands.	34% women	Non-small cell lung cancer.	-
					Mean age: 66 years	Stage IV: 24%	
Medical Care Questionnaire (MCQ) [41]	Phase	Phase 3:	Adult patients from all tumor groups attending the Medical Oncology Unit, could read and understand English, were not exhibiting overt cognitive dysfunction or signs of distress.	A regional hospital, England.	Phase 3:	Phase 3:	-
	3: 200	70%			81% women	Gynecological: 38%	
	Phase 4: 477	Phase 4: 79.6%			42% aged 45-59 years	Breast: 26.5%	
					74% married/de facto	Genitourinary: 16.5%	
					13% employed full time	Sarcoma: 5.5%	
						Gastrointestinal: 4.5%	
					Phase 4:	Melanoma: 1%	
					74.2% women	Other: 8%	
					40.9% aged 45-59 years		
					75.1% married/de facto	Phase 4:	
					44.2% employed full time	Gynecological: 33.8%	
						Breast: 23.5%	
						Genitourinary: 21.4%	
						Melanoma: 8.4%	
						Sarcoma: 7.5%	
						Other: 5.5%	
						-	
Modified Version of the Perceived Involvement in Care Scale (M-PICS) [42]	87	74%	Females aged 18 years or older, confirmed diagnosis of breast cancer, reported pain of at least moderate intensity (score ≥4 on the Brief Pain Inventory’s Worst Pain Intensity item) over prior two weeks, absence of any gross cognitive impairment, literate in English or Spanish.	Four hospital-based outpatient oncology clinics and a private hospital-affiliated oncology practice, USA.	100% women	Breast cancer.	89.7% chemotherapy
					Mean age: 50.4 years	Stage I: 12.6%	43.7% radiation therapy
					31% Caucasian	Stage II: 24.1%	
					50.6% married/partnered	Stage III: 13.8%	
					64.5% at least 13 years education	Stage IV: 49.4%	
					26.2% employed		
Oncology Patients’ Perceptions of the Quality of Nursing Care Scale (OPPQNCS) [43]	436	-	18 years or older, registered with the receptionist on the days of data collection, had received cancer nursing care in the clinic or hospital, not extremely ill or confused.	A haematology-oncology clinic, USA.	66% women	Breast: 40%	In active treatment
					Mean age: 54.8 years	Melanoma: 9%	
					93% white	Lung: 6%	
					81% more than high school education	Renal cell: 4%	
						Squamous cell: 4%	
						Prostate: 3%	
						Other: <3%	
						-	
Pain Care Quality Survey (PainCQ) [44,45]	109	-	18+ years of age, inpatients on one of the designated units with an expected stay of more than 24 hours, diagnosis of cancer, surgery for cancer, a suspected cancer diagnosis or a hematological disorder and a positive response to screening regarding the presence of pain, cognitively and physically able to complete survey.	Hospitals in three geographically diverse settings with medical or surgical oncology units, USA.	58.7% women	Leukaemia/lymphoma: 15.6%	Reason for hospitalization:
					Mean age: 53.1 years		
					88.1% non-Hispanic white	Uterine/Cervical/Ovarian: 11.9%	Surgery: 40.4%
							Supportive care and management of complications: 45.9%
					66.1% married/partnered	Prostate and genitourinary: 11.9%	
					25.7% college graduate		
						Colorectal: 10.1%	Treatment of cancer: 8.3%
						Lung: 7.3%	
						Gastrointestinal: 7.3%	Other: 5.5%
						Breast: 6.4%	
						Other cancers: 22.0%	
						Non-cancer diagnosis: 7.3%	
						34.9% local or regional	
						41.3% advanced cancer	
PASQOC questionnaire [46]	2659	78.6%	Aged 18 years or older, the presence of any cancer suitable for outpatient treatment, read and write in German, mentally and physically able to complete questionnaire.	24 institutions including 15 private group practices, 6 single oncologist-led practices, 3 hospital day clinics, Germany.	56% women	Breast: 22.9%	80.9% chemotherapy
					Mean age: 61.7 years	Intestine: 19.8%	58.0% surgery
					8.7% employed full-time	Lymphoma: 15.2%	
						Haematological: 12.3%	
						Other: 29.8%	
						58.2% distant metastases	
						61.9% diagnosed within last 3 years	
Patient Satisfaction with Cancer Care [47]	891	-	Abnormal breast, cervical, colorectal and prostate cancer test finding or a new diagnosis of these cancers without any prior history of cancer treatment other than non-melanoma skin cancer, fluent in English.	Multiple patient Navigation Research Program recruitment sites (eg, clinics or hospitals), within nine largely racial/ethnic minority and low-income communities, USA.	81.3% women	Breast: 64.2%	-
					Mean age: 51.4 years	Cervix: 10.8%	
					43.2% white	Colorectal: 12.0%	
					40.4% married/defacto	Prostate: 12.6%	
					12.9% college graduate	Multiple sites: 0.5%	
					29.9% full-time employed		
						-	
Perceived Physician’s Communication Style Scale [48]	147	74%	Patients with cancer who were aware of their cancer diagnosis, met their doctor more than once and did not have a debilitating condition.	A hospital, Japan.	66.7% women	Breast cancer: 50.3%	-
					Mean age: 57.6 years	Gastric cancer: 30.6%	
					20.4% university educated	Lung cancer: 15.0%	
						Other cancer: 4.1%	
						55.1% disease free	
						44.9% recurrent or metastatic disease	
Prostate Care Questionnaire for Patients (PCQ-P) [49]	865	69.2%	Patients diagnosed with, or treated for prostate cancer within the past two years, who were not too ill to participate.	5 hospitals, England	100% male	Prostate cancer	-
					40.5% aged 65-74 years		
					92.8% white	-	
					21.3% employed		
QUOTE Breast Cancer [50,51]	276	43%	Experience with any type of surgery for breast cancer 3 -15 months before the start of the study, age older than 17 years and mental competence as judged by the breast nurse.	5 hospitals, The Netherlands.	100% women	Breast cancer	54% lumpectomy
					Mean age: 57 years		52% (modified) radical mastectomy
					32% college/university educated	16% diagnosed 3-6 months ago, 47% 7-12 months ago, 34% 13-18 months ago, 3% more than 18 months ago.	50% radiotherapy
							38% chemotherapy
QUOTE^chemo^[52]	345	59.3%	60 most recent patients from each hospital who were new to chemotherapy, aged 18 years or older, able to read Dutch.	Hospital records from 10 hospitals, The Netherlands.	67% women	Breast: 47.2%	16.2% chemotherapy only
					Mean age: 55.7 years	Digestive-gastrointestinal: 21.5%	
					79% lived with partner		74.5% chemotherapy & surgery
					28% highly educated	Haematologic: 10.6% Lung: 9.7%	
					52.8% employed	Gynaecological: 6.2% Urologic: 2.9%	45.5% chemotherapy & radiotherapy
						Other: 1.8%	21.1% chemotherapy & hormone replacement therapy
						Mean of 11.5 months since diagnosis.	6.2% chemotherapy & immunotherapy
							74.8% curative intent
							25.2% palliative intent
REPERES-60 [53]	820	84%	A first diagnosis of invasive non-metastatic breast cancer, at least two contacts for cancer with one of the health professionals in one of the two regions between diagnosis and the first year of follow-up, signed informed consent.	Public and private cancer centres in two regions, France.	100% women	Breast cancer.	-
					Mean age: 58 years		
					55.1% living with spouse/partner	-	
					15.7% higher education 43.7% employed		
Satisfaction with Cancer Information Profile (SCIP) [54]	82	76%	Newly diagnosed patients with head and neck cancer.	4 hospitals, England.	34% women	Head and neck cancer. Most common sites tongue and laryngeal/glottis	Planned treatments:
							27% surgery only
							26% radiotherapy only
							31% surgery and radiotherapy
					Mean age: 60 years	Stages I and II: approximately 50%; Stages III and IV: approximately 50%	11% radiotherapy and chemotherapy
					92% white 61% married/de facto		5% surgery, radiotherapy and chemotherapy
SAT-RAR [55]	297	55.8%	Aged > 18 years, curative irradiation and satisfactory general status (<3 on WHO performance scale).	16 centers, France.	Breast cancer (n = 98):	Non-small cell lung cancer.	100% radiotherapy
					Mean age: 56 years		Respiratory gating: 44% (breast cancer) & 67% (lung cancer)
					100% women	Breast cancer.	
					Mean days hospitalized: 4.7 days	-	
					Non-small cell lung cancer (n = 199):		1 or more acute toxicity during treatment: 96% (breast cancer) & 86% (lung cancer)
					Mean age: 65 years 15% women		
					Mean days hospitalized: 3.8 days Mean number of hospitalizations: 7.8		

### Patient-centred care instruments

The names of the PROMs included in the review are shown in Tables 1, 2, 3, 4 and 5. As shown in Table 2, 15 measures examined patients’ experiences of care [32,33,37,39-44,48-50,52,53,55] while 6 measured satisfaction [34,35,38,46,47,54]. The number of items for each measure ranged from 15 to 152, and the number of subscales ranged from 1 to 15. The type of response scales varied across the different instruments. The number of IOM-endorsed patient-centred care dimensions [1] that were included in each measure were as follows: two measures included one dimension [35,54], two measures had two dimensions [42,46], seven measures had three dimensions [34,39,41,47,48,50,55], five measures had four dimensions [32,33,37,49,53], and four measures had five dimensions [38,43,44,52]. Only one measure, the Indicators (Non-small Cell Lung Cancer) measure, covered all six dimensions of patient-centred care [40]. Table 3 summarises the PROMs that addressed each of the IOM-endorsed patient-centred care dimensions.

**Table 2:** Measurement features of PROMs and included IOM-endorsed patient-centred care dimensions

**Measure**	**Satisfaction/experience**	**Number of items**	**Response scale**	**Subscales**	**IOM patient-centred dimensions**
Assessment of Patient Experiences of Cancer Care (APECC) [32]	Experiences	33	Not a problem, A small problem, A big problem.	Getting needed care	Emotional support
				Timeliness of care	Information & communication
			Never, Sometimes, Usually, Always.	Waiting time in physician’s office	Integrated & coordinated care
				Information exchange	Respectful to patients’ values
				Physicians’ affective behavior	
				Physicians’ knowledge	
			On time, < 15 minutes, 16-30 minutes, 31-45 minutes, > 45 minutes.	Interaction with nurses	
				Interaction with office staff	
				Health promotion	
				Coordination of care	
			Poor, Fair, Good, Very good, Excellent.	Overall rating of care	
			Yes definitely, Yes somewhat, No.		
			0 (worst doctor possible) to 10 (best doctor possible)		
			Definitely yes, Probably yes, Not sure, Probably not, Definitely not.		
Cancer Care Coordination Questionnaire for Patients [33]	Experiences	20	Strongly disagree, Disagree, Neutral, Agree, Strongly agree.	Communication	Emotional support
				Navigation	Family & friends
			Never, Rarely, Sometimes, Frequently, Always.		Information & communication
					Integrated & coordinated care
Cancer Patient Information Importance and Satisfaction Tool [34]	Satisfaction	24	5 point scale from Not important (0) to Very important (4).	Information importance	Emotional support
				Information satisfaction	Information & communication
					Physical comfort
			5 point scale from Not satisfied (0) to Very satisfied (4).		
Cancer Therapy Satisfaction Questionnaire (CTSQ) [35,36]	Satisfaction	16	5 point scale with 1 representing the worst response and 5 representing the best response.	Expectation of therapy	Respectful to patients’ values
				Feelings about side effects	
				Satisfaction with therapy	
Consumer Quality Index Breast Care (CQI-BC) [37]	Experiences	152 (118 items related to patients’ experiences)	Never, Sometimes, Usually, Always.	Conduct of professionals during breast examination	Emotional support
					Information & communication
			Yes, No.	Conduct of general practitioner	Integrated & coordinated care
				Conduct of nurses	Respectful to patients’ values
			A big problem, A small problem, No problem.	Conduct of surgeon	
				Autonomy regarding treatment	
				Autonomy regarding follow-up treatment	
				Conduct of professionals during radiotherapy	
				Information on radiotherapy	
				Conduct of professionals during chemotherapy	
				Information on chemotherapy	
				Cooperation	
				Accessibility of care	
				Continuity psychosocial care	
				Continuity physiotherapy	
				Continuity rehabilitation	
EORTC cancer in-patient satisfaction with care measure (EORTC IN-PATSAT32) [38]	Satisfaction	32	Poor, Fair, Good, Very good, Excellent.	Doctors’ technical skills	Emotional support
				Doctors’ interpersonal skills	Information & communication
				Doctors’ information provision	Integrated & coordinated care
				Doctors’ availability	Physical comfort
				Nurses’ technical skills	Respectful to patients’ values
				Nurses’ interpersonal skills	
				Nurses’ information provision	
				Nurses’ availability	
				Exchange of information	
				Other hospital staff interpersonal skills and information provision	
				Waiting time	
				Hospital access	
				Comfort	
				General	
				satisfaction	
Indicators (Head & Neck Cancer) [39]	Experiences	23 specific indicators for patients	-	Patient-oriented quality of care	Emotional support
				Organisational quality of care	Information & communication
				Medical/technical quality of care	Integrated & coordinated care
Indicators (Non-small Cell Lung Cancer) [40]	Experiences	56	1 = Not done, 2 = Done, but inadequately, 3 = Done adequately, 4 = Done excellently.	Access	Emotional support
				Follow up	Family & friends
				Communication and respect	Information & communication
				Patient & family involvement	Integrated & coordinated care
			Yes, No	Information	Physical comfort
				Coordination	Respectful to patients’ values
				Physical support	
				Emotional & psychosocial support	
Medical Care Questionnaire (MCQ) [41]	Experiences	15	-	Communication	Emotional support
				Preferences	Integrated & coordinated care
				Coordination	Respectful to patients’ values
Modified Version of the Perceived Involvement in Care Scale (M-PICS) [42]	Experiences	20	1 = All the time to 5 = Never.	Health care provider information	Information & communication
				Patient information	Respectful to patients’ values
				Patient decision making	
				Health care provider facilitation	
Oncology Patients’ Perceptions of the Quality of Nursing Care Scale (OPPQNCS) [43]	Experiences	40 (and 18-item short form created)	1 = Never to 6 = Always, Didn’t matter, Don’t know.	Responsiveness	Emotional support
				Individualization	Family & friends
				Coordination	Information & communication
				Proficiency	Integrated & coordinated care
					Respectful to patients’ values
Pain Care Quality Survey (PainCQ) [44,45]	Experiences	33	1 = Strongly disagree to 6 = Strongly agree.	*PainCQ-Interdisciplinary scale:*	Family & friends
				Partnership with healthcare team	Information & communication
				Comprehensive interdisciplinary pain care	Integrated & coordinated care
					Physical comfort
				*PainCQ-Nursing scale:*	Respectful to patients’ values
				Being treated right	
				Comprehensive nursing pain care	
				Efficacy of pain management	
PASQOC questionnaire [46]	Satisfaction	120	Nominal or ordinal scales and some interval scales	5 dimensions reported	Information & communication
				Patient-provider relationship	Respectful to patients’ values
				Premises	
				Information on diagnosis & treatment	
				Information on treatment consequences	
				Relationship between patient & nurse	
Patient Satisfaction with Cancer Care [47]	Satisfaction	18	1 = Strongly Agree to 5 = Strongly Disagree	1 component structure – satisfaction with cancer care	Information & communication
					Integrated & coordinated care
					Respectful to patients’ values
Perceived Physician’s Communication Style Scale [48]	Experiences	27	1 = Strongly Disagree, 3 = Neutral, 5 = Strongly Agree.	Acceptive	Emotional support
				Patient-centered	Information & communication
				Attentive	Respectful to patients’ values
				Facilitative	
Prostate Care Questionnaire for Patients (PCQ-P) [49]	Experiences	106 (Sections A-E).	Various scales – please see reference [63]	*Information from additional file 1*	Information & communication
				*Section A: GP visits and referral*	Integrated & coordinated care
				Explanation	Physical comfort
				Experience of referral	Respectful to patients’ values
				Taking the problem seriously	
				*Section B: Tests at the hospital*	
				Explanation & support	
				Quality of care	
				Appointment	
				*Section C: Diagnosis and treatment decision*	
				Explanation & support	
				Making treatment decision	
				Getting the diagnosis	
				Length of wait	
				*Section D: Treatment and discharge*	
				Preparation for discharge	
				Treatment	
				Information	
				*Section E: Monitoring*	
				Explanation & reassurance	
				Advice	
				Choice	
QUOTE Breast Cancer [50,51]	Experiences	33	*Performance:*	Patient education regarding aspects related to postoperative treatment	Information & communication
			Never, Sometimes, Usually, Always.		Integrated & coordinated care
				Services by the breast nurse	Respectful to patients’ values
			Yes, No.	Services by the surgeon	
				Patient education regarding activities at home	
			Not applicable/I do not know added to a subset of items.	Patient education regarding aspects related to preoperative treatment	
			*Importance:*		
			Not important, Fairly important, Important, Extremely important.		
QUOTE^chemo^[52]	Experiences	67	*Performance:*	Treatment-related information	Emotional support
			Yes, No.	Prognosis information	Family & friends
			*Importance:*	Rehabilitation information	Information & communication
				Coping information	Physical comfort
			Not important, Fairly important, Important, Very important.	Interpersonal communication	Respectful to patients’ values
				Tailored communication	
				Affective communication	
REPERES-60 [53]	Experiences	60	Bad, Fair, Good, Very good, Excellent.	Access to primary care	Emotional support
				Access to secondary care	Information & communication
		
			Completely agree, Agree generally, No marked opinion, Do not really agree, Do not agree at all.	Competence and communication skills of primary care doctors	Integrated & coordinated care
				Competence of secondary care doctors	Respectful to patients’ values
				Communication skills of secondary care doctors	
			Bad, Fair, Good, Very good, Excellent, Not concerned.		
				Choice among different doctors	
				Human qualities of doctors	
				Global satisfaction	
				Cover for medical expenses	
				Listening abilities and information provided by doctors	
				Organisation and follow-up of medical care provision	
				Psychological support	
				Material environment	
Satisfaction with Cancer Information Profile (SCIP) [54]	Satisfaction	21	Too much, About right, Too little, None wanted.	Satisfaction with the amount and content of information	Information & communication
			Very satisfied, Satisfied, Neither, Dissatisfied, Very dissatisfied.	Satisfaction with the form and timing of the information received	
SAT-RAR [55]	Experiences	23	Poor, Fair, Good, Very good, Excellent.	Perception of the radiotherapist or radiotherapy technicians	Emotional support
					Information & communication
			Not at all, A little, Quite a bit, Very much.	Global satisfaction	Physical comfort
				Treatment experience	
			Poor, Moderate, Good, Very good, Excellent.		
			Disagree, Unsure, Tend to agree, Agree, Strongly agree.		

**Table 3:** IOM patient-centred care dimensions captured by PROMs

**Measure**	**IOM patient-centred care dimensions**					
	**Emotional support**	**Family & friends**	**Information & communication**	**Integrated & coordinated care**	**Physical comfort**	**Respectful to patients’ values**
APECC [32]	√		√	√		√
Cancer Care Coordination Questionnaire for Patients [33]	√	√	√	√		
Cancer Patient Information Importance and Satisfaction Tool [34]	√		√		√	
CTSQ [35,36]						√
CQI-BC [37]	√		√	√		√
EORTC IN-PATSAT32 [38]	√		√	√	√	√
Indicators (Head & Neck Cancer) [39]	√		√	√		
Indicators (Non-small Cell Lung Cancer) [40]	√	√	√	√	√	√
MCQ [41]	√			√		√
M-PICS [42]			√			√
OPPQNCS [43]	√	√	√	√		√
PainCQ [44,45]		√	√	√	√	√
PASQOC questionnaire [46]			√			√
Patient Satisfaction with Cancer Care [47]			√	√		√
Perceived Physician’s Communication Style Scale [48]	√		√			√
PCQ-P [49]			√	√	√	√
QUOTE Breast Cancer [50,51]			√	√		√
QUOTE^chemo^[52]	√	√	√		√	√
REPERES-60 [53]	√		√	√		√
SCIP [54]			√			
SAT-RAR [55]	√		√		√	

**Table 4:** Psychometric properties of PROMs assessing quality of patient-centred cancer care

**Measure**	**Face validity/content validity**	**Construct validity**			**Internal consistency**	**Test-retest reliability**	**Cross-cultural adaptation**
		**Factor analysis**	**Known groups**	**Existing measure**			
Assessment of Patient Experiences of Cancer Care (APECC) [32]	All items underwent cognitive testing with nine cancer survivors to ensure that the questions and response options were understandable and related to the concept being measured.	Confirmatory factor analysis indicated a reasonably good fit for the 10-factor model (comparative fit index = 0.93).	-	-	Getting needed care: α =.76	-	-
					Timeliness of care: α =.62		
					Waiting time in physician’s office: α =.65		
					Information exchange: α =.92		
					Physicians’ affective behavior: α =.92		
					Physicians’ knowledge: α =.86		
					Interaction with nurses: α =.82		
					Interaction with office staff: α =.90		
					Health promotion: α =.88		
					Coordination of care: N/A		
					Overall rating of care: α=.87		
Cancer Care Coordination Questionnaire for Patients [33]	Literature review undertaken to identify relevant issues and existing instruments and focus groups and semi-structured interviews with 24 cancer patients and carers and 29 clinicians. Draft questionnaire was reviewed by clinicians and researchers to assess face validity and clarity of wording.	Exploratory factor analysis. Principal factor method followed by a promax rotation.	-	-	Total scale: α=.88	Sample 1: 119 patients completed the survey twice -mailed 2 weeks after receipt of first survey.	-

					Communication: α=.87		
					Navigation: α=.73		
		Factor loadings >.40 with the exception of one item (0.37).				Kappa for individual items ranged from 0.29 to 0.69. Four items with values less than 0.40 were eliminated.	
		Eigenvalues>1					
Cancer Patient Information Importance and Satisfaction Tool [34]	Literature review and extensive qualitative interviews with cancer patients. Tool was field-tested with 10 cancer patients who completed tool and provided feedback about its clarity and ease of completion.	-	-	-	Information importance: α=.89	-	-
					Information satisfaction: α=.92		
Cancer Therapy Satisfaction Questionnaire (CTSQ) [35,36]	Interviews with 70 oncology patients, 4 oncology nurses and 7 physicians. Focus groups with 14 oncology nurses. Content validity tested with 30 patients who completed the survey and were interviewed, followed by retesting in an additional 10 patients.	Exploratory factor analysis using oblique promax rotation.	Cancer stage (I, II, III, IV) *P* <0.001 for 1 subscale.	Treatment Satisfaction Questionnaire for Medication 6 correlations > .40	Expectations of therapy: α=.87	85 patients completed follow-up questionnaires one week after baseline assessment.	-
					Feelings about side effects: α=.77	Intraclass correlation:	
					Satisfaction with therapy: α=.82	Expectations of therapy: .56	
			Side effects (with, without) *P*<0.05 for 2 subscales.	EORTC QLQ-C30 1 correlation >.40		Feelings about side effects: .77	
			ECOG performance status (Grade 0, 1, 2, 3) *P*<0.005 for 1 subscale.			Satisfaction with therapy: .75	
Consumer Quality Index Breast Care (CQI-BC) [37]	Three focus groups with 27 breast cancer patients, existing Dutch questionnaires on breast care and key stakeholders’ input used for questionnaire development.	Explorative factor analysis.	-	-	Conduct of professionals during breast examination: α=.91	-	-
					Conduct of general practitioner: α=.89		
					Conduct of nurses: α=.88		
					Conduct of surgeon: α=.91		
					Autonomy regarding treatment: α=.84		
					Autonomy regarding follow-up treatment: α=.93		
					Conduct of professionals during radiotherapy: α=.89		
					Information on radiotherapy: α=.89		
					Conduct of professionals during chemotherapy: α=.90		
					Information on chemotherapy: α=.85		
					Cooperation: α=.88		
					Accessibility of care: α=.68		
					Continuity psychosocial care: α=.83		
					Continuity physiotherapy: α=.82		
					Continuity rehabilitation: α=.80		
EORTC cancer in-patient satisfaction with care measure (EORTC IN-PATSAT32) [38]	Adapted from existing patient satisfaction questionnaires (Comprehensive Assessment of Satisfaction with Care and EORTC QLQ-SAT32) [64,65], as well as interviews with oncology specialists and cancer patients.	-	Age (less than 57 years, 57 years or more) *P* <0.05 for 4 subscales.	Oberst Patients’ Perception of Care Quality and Satisfaction Scale correlations (-0.21 to -0.61).	Doctors’ technical skills: α=.85−.87	113 patients recruited from one centre for follow-up approximately 2 weeks after first assessment.	Validated with Sri Lankan cancer patients (n=343) [56]
			Education (less than compulsory, post-compulsory) *P* <0.05 for 2 subscales.		Doctors’ interpersonal skills: α=.91−.94		
					Doctors’ information provision: α=.90−.94		
					Doctors’ availability: α=.86−.91		
					Nurses’ technical skills: α=.90−.94		
					Nurses’ interpersonal skills: α=.90−.93	Intra-class correlations coefficients for the scales ranged from 0.70-0.85 and was 0.66 for the general satisfaction item.	
					Nurses’ information provision: α=.94−.96		
					Nurses’ availability: α=.83−.92		
			Treatment related toxicity (yes, no) *P* <0.05 for 7 subscales.	EORTC QLQ-C30 r<.30.	Exchange of information: Ν/Α		
					Other hospital staff interpersonal skills and information provision: α=.86−.90		
					Waiting time: α=.80−.84		
					Hospital access: α=.56−.71		
					Comfort: Ν/Α		
					General satisfaction: N/A		
Indicators (Head & Neck Cancer) [39]	Systematically searched for recommendations in literature, performed a systematic consensus procedure based on evidence-based guidelines and sought opinions of 15 professionals and 30 patients with head and neck cancer.	-	-	-	-	-	-
Indicators (Non-small Cell Lung Cancer) [40]	Recommendations for patient-centred care extracted from clinical guidelines and conducted semi-structured interviews with 30 head and neck cancer patients and 7 patient representatives from the Dutch national association of patients with lung cancer. Two researchers translated recommendations into indicators which were considered by a panel of four researchers.	-	-	-	Access: α=.87	-	-
					Follow up: α=.78		
					Communication and respect: N/A		
					Patient & family involvement: α=.85		
					Information: α=.78		
					Coordination: α=.22 (specialists) & α=.68 (oncology nurses)		
					Physical support: N/A		
					Emotional & psychosocial support: α=.67		
Medical Care Questionnaire (MCQ) [41]	Literature review of existing instruments, modification (items removed, reworded, generated) of an existing instrument by an expert panel (3 medical oncologists and one oncologist in training) using a consensus procedure, instrument administered to 200 oncology outpatients and then refined.	Exploratory factor analysis using oblique rotation (Phase 3) and confirmatory factor analysis (Phase 4).	Cancer type (Breast, Genitourinary, Gynecological, Melanoma, Sarcoma, Other) *P* <0.05 for 3 subscales.	-	Communication: α=.69	-	-
		Factor loadings >.40			Preferences: α=.84		
		Eigenvalues > 1			Coordination: α=.75		
Modified Version of the Perceived Involvement in Care Scale (M-PICS) [42]	Literature review and consultation with pain clinicians guided augmentation and addition of items on the original Perceived Involvement in Care Scale (PICS).	Exploratory factor analysis – principal components analysis with oblique rotation.	Age *P*<0.01 for 1 subscale.	Barriers Questionnaire-II	Total scale: α= .87	-	Validated with Lithuanian cancer patients (n=30) [57]
		Factor loadings >.40	Ethnicity (Latina; Caucasian/African- American) *P*<0.01 for 2 subscales.	2 correlations >.40	Health care provider information: α= .90		
				Mental Health Inventory	Patient information: α= .82		
				3 correlations <.30	Patient decision making: α= .80		
				Medical Outcomes Study Short-Form 12: Mental Component Scale	Health care provider facilitation: α= .80		
				3 correlations <.30			
				Medical Outcomes Study Short-Form 12: Physical Component Scale			
				4 correlations <.30			
				Patient Satisfaction Questionnaire			
				3 correlations >.40			
Oncology Patients’ Perceptions of the Quality of Nursing Care Scale (OPPQNCS) [43]	Interviews with cancer patients about their perceptions of nursing care, items and subscales generated from this qualitative work, an expert methods consultant evaluated items for clarity and relevance and a nine-member expert rater panel consisting of 5 cancer patients, a nurse, a nurse researcher, the executive director of a patient advocacy group and a survey scientist, reviewed and reduced items.	Exploratory factor analysis – principal components analyses with promax (oblique) rotation.	-	-	Total scale: α= .99	-	Validated with Turkish cancer patients (n=54) [58]
					Responsiveness: α= .99		

					Individualization: α= .97		
					Coordination: α= .87		
		Factor loadings >.40			Proficiency: α= .95		
Pain Care Quality Survey (PainCQ) [44,45]	Conducted 33 qualitative interviews with cancer patients in pain. Items were constructed using this qualitative work, existing tools, recommendations for item development in the literature, and consultation with a national expert in tool development. Two panels of pain and quality experts reviewed items (removed, reworded or added items). Cognitive interviews were then undertaken with 39 hospitalized cancer patients reporting pain.	Exploratory factor analysis – principal axis factoring using an oblimin rotation.	-	-	*PainCQ-Interdisciplinary scale:*	-	-
					Partnership with healthcare team: α=.85		
					Comprehensive interdisciplinary pain care: α=.76		
					*PainCQ-Nursing scale:*		
					Being treated right: α=.95		
		Factor loadings >.40			Comprehensive nursing pain care: α=.77		
		Eigenvalues > 1			Efficacy of pain management: α=.87		
PASQOC questionnaire [46]	Existing survey re-designed and content similar to other surveys. Focus group discussions with 29 patients in four centres.	Factor analysis	-	Short Form-36 (SF-36) correlations (.042 to -.161)	Total scale: α= .93	-	-
	Pre-testing the questionnaire for appropriateness of the questions and length with 280 patients from 14 centres.	Factor loadings >.40 with the exception of one item (.394).			Patient-provider relationship: α=.81		
					Premises: α=.76		
					Information on diagnosis & treatment: α=.71		
					Information on treatment consequences: α=.87		
					Relationship between patient & nurse: α=.72		
Patient Satisfaction with Cancer Care [47]	Item pool based on literature review and existing measures, expert feedback, group discussion and consensus.	Factor analysis – principal components analysis	-	Communication and Attitudinal Self-Efficacy – Cancer 1 correlation >.40 (sample 2).	Total scale: α= .95 and .96 (for two samples).	-	-
		Factor loadings >.40					
		Eigenvalue >1					
Perceived Physician’s Communication Style Scale [48]	Initial item pool created from literature review and 25 cancer patients’ opinions about the physician’s communication style.114 nurses assessed the content validity of items. Items pre-tested with 70 breast cancer patients.	Factor analysis using varimax procedure.	-	-	Total scale: α= .95	-	-
		Factor loadings >.40			Acceptive: α=.90		
		Eigenvalues > 1			Patient-centered: α=.90		
					Attentive: α=.73		
					Facilitative: α=.76		
Prostate Care Questionnaire for Patients (PCQ-P) [49]	Initial items developed through a literature review and interviews with patients and service providers. Semi-structured interviews with 20 prostate cancer patients who completed survey.	Exploratory principal components analysis with varimax rotation.	-	National Centre for Social Research Shortened Questionnaire	Section A: α=.80	148 (50%) patients from two hospitals completed retest survey mailed 3 weeks later.	-
		Factor loadings for each section of 0.3 and higher presented in an additional file.		Sections B & C	Section B: α=.63		
				r <.30	Section C: α=.77		
				Sections D & E r >.40	Section D: α=.80		
					Section E: α=.68	Intraclass correlation coefficient:	
						Section A: .68	
						Section B: .57	
						Section C: .61	
						Section D: .73	
						Section E: .70	
						Identical responses to individual questions: 52.6% to 100%	
QUOTE Breast Cancer [50,51]	Based on eight focus groups with 72 breast cancer patients and concept mapping sessions with 67 breast cancer patients a pilot questionnaire was developed. Two researchers categorised and reduced the aspects of care identified.	Exploratory factor analysis (i.e., principal axis factoring) – oblique rotation.	Age (18-49, 50-65, >65 years) no difference.	-	Patient education regarding aspects related to postoperative treatment: α=.83	-	-
		Factor loadings >.40 except for 0.35 loading and 6 separate items.			Services by the breast nurse: α=.89		
		Eigenvalues >1	Education (primary school, secondary school, college/university) no difference.		Services by the surgeon: α=.85		
					Patient education regarding activities at home: α=.70		
			Time since surgery (0-6, 6-12, >12 months) no difference.		Patient education regarding aspects related to preoperative treatment: α=.81		
QUOTE^chemo^[52]	Items developed via existing measure, literature review and 5 focus groups (n=33) as well as individual interviews with 5 cancer patients. 10 coders categorised the items into the seven dimensions.	Confirmatory factor analysis.	-	QUOTE^chemo^ Importance with:	*Performance:*	-	-
		Factor loadings >.40			Treatment-related information: α=.92		
					Prognosis information: α= .72		
					Rehabilitation information: α= .87		
				Information Satisfaction Questionnaire	Coping information: α= .78		
				7 correlations r <.30	Interpersonal communication: α= .89		
					Tailored communication: α= .86		
					Affective communication: α= .91		
				Threatening Medical Situation Inventory	*Importance:*		
				7 correlations r <.30	Treatment-related information: α=.90		
				Impact of Event Scale:	Prognosis information: α= .76		
				- Intrusion 7 correlations r <.30	Rehabilitation information: α= .86		
				- Avoidance 7 correlations r <.30	Coping information: α= .81		
					Interpersonal communication: α= .90		
					Tailored communication: α= .81		
					Affective communication: α= .88		
REPERES-60 [53]	Literature review and two focus groups with 30 breast cancer patients used to generate items and identify domains. Adapted existing Consumer Satisfaction Survey and developed new items based on patient focus groups and experts’ input. Test version of questionnaire tested with breast cancer patients to assess understanding and comprehensiveness of items, which led to minor alterations.	Principal components factor analysis (varimax rotation).	Age (less than 58 years, 58 years or older)	EORTC QLQ-C30 r <.30	Access to primary care: α=.88	166 (91%) patients sent retest 1 week later.	-
		Factor loadings >.40	*P* <0.05 for 11 subscales.		Access to secondary care: α=.82		
		Eigenvalues >1	Education (primary and secondary, higher education)		Competence and communication skills of primary care doctors: α=.93	Item-to-item agreement:	
			*P* <0.05 for 8 subscales.		Competence of secondary care doctors: α=.94	Kappa ranged from 0.44 to ≥ 0.70	
					Communication skills of secondary care doctors: α=.92		
					Choice among different doctors: α=.87		
					Human qualities of doctors: α=.94		
					Global satisfaction: α=.74		
					Cover for medical expenses: α=.90		
					Listening abilities and information provided by doctors: α=.93		
					Organisation and follow-up of medical care provision: α=.87		
					Psychological support: α=.88		
					Material environment: α=.89		
Satisfaction with Cancer Information Profile (SCIP) [54]	Four items derived from Satisfaction with Information About Medicines Scale (SIMS) and additional items from patient interviews.	-	-	Illness Perception Questionnaire-Revised	Satisfaction with the amount and content of information: α=.89	-	-
				2 correlations r <.30	Satisfaction with the form and timing of information: α=.87		
SAT-RAR [55]	Review of the literature, selection and formation of items based on relevant themes and a cancer care satisfaction questionnaire, pilot-testing of preliminary questionnaire with 10 patients to evaluate relevance, content validity and comprehensibility of items, survey reviewed by clinicians in the study.	Exploratory factorial analysis with varimax rotation followed by Confirmatory factorial analysis.	Education (at least high school completed, high school not completed)	-	Total scale: α=.86	-	-
			*P* <0.01 in 1 subscale.		Perception of the radiotherapist or radiotherapy technicians: α=.94		
		Most factor loadings >.40, except 3 factor loadings >.30	Marital status (married, not married)		Global satisfaction: α=.80		
			*P* <0.01 in 1 subscale.		Treatment experience: α=.75		
			Work status (employed, not employed)				
			*P* <0.01 in 2 subscales.				
			Type of radiotherapy (respiratory gating, no respiratory gating)				
			*P* <0.01 in 2 subscales.				

**Table 5:** PROMs demonstrating adequate psychometric properties based on recommended criteria

**Measure**	**Face/content validity**	**Construct validity**			**Internal consistency**	**Test-retest reliability**	**Cross-cultural adaptation**
		**Factor analysis**	**Known groups**	**Existing measure**			
APECC [32]		√					
Cancer Care Coordination Questionnaire for Patients [33]	√	√			√		
Cancer Patient Information Importance and Satisfaction Tool [34]							
CTSQ [35,36]	√	√	√	√			
CQI-BC [37]	√	√					
EORTC IN-PATSAT32 [38]	√		√	√			√
Indicators (Head & Neck Cancer) [39]	√						
Indicators (Non-small Cell Lung Cancer) [40]	√						
MCQ [41]	√	√	√				
M-PICS [42]		√	√	√	√		√
OPPQNCS [43]	√	√			√		√
PainCQ [44,45]	√	√					
PASQOC questionnaire [46]		√		√	√		
Patient Satisfaction with Cancer Care [47]		√		√	√		
Perceived Physician’s Communication Style Scale [48]	√	√			√		
PCQ-P [49]	√	√		√			
QUOTE Breast Cancer [50,51]	√	√					
QUOTE^chemo^[52]	√	√		√			
REPERES-60 [53]	√	√	√	√			
SCIP [54]				√			
SAT-RAR [55]	√	√	√		√		

**Figure 2:**
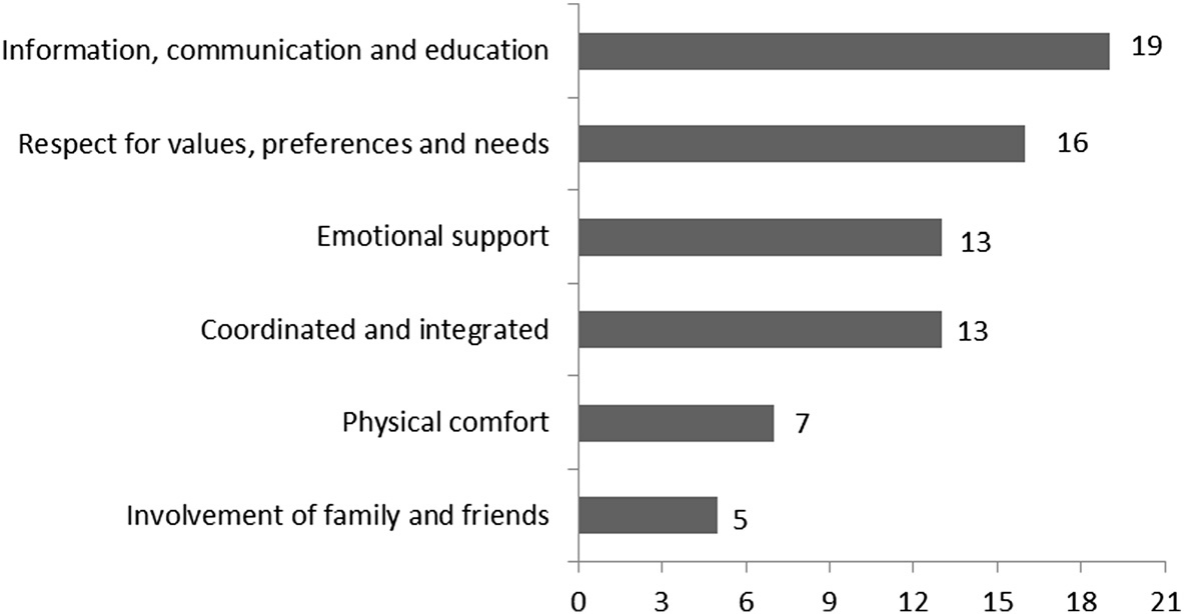
Frequency of IOM-endorsed patient-centred care dimensions across 21 measures.

Figure 2 illustrates the frequency with which the six IOM-endorsed patient-centred dimensions were included across the 21 measures. “Information, communication and education” was the dimension most commonly included (19 measures). In contrast, only five measures assessed the “involvement and wellbeing of family and friends”. Thirteen measures addressed *all* the IOM criteria for the emotional support dimension [32-34,37-41,43,48,52,53,55], 8 measures for information, communication and education [32,37,47,48,50,52,54,55] and one measure for physical comfort [44]. None of the measures addressed all the IOM criteria within the dimensions of respect for patient values, preferences and needs; coordinated and integrated care; and involvement and wellbeing of family and friends.

### Psychometric properties of instruments

A description of the psychometric properties for each PROM is reported in Table 4.

#### Internal consistency

Seven of the 21 measures met the criteria considered adequate for internal consistency by reporting a Cronbach’s alpha >0.70 for *both* the total scale and each sub-scale [33,42,43,46-48,55]. Of the 13 studies that reported Cronbach’s alpha only for the PROMs’ subscales, six of these measures showed all subscales had a Cronbach’s alpha >0.70 [34,35,44,52-54].

#### Test-retest reliability

None of the five measures that examined test-retest reliability [33,35,38,49,53] met recommended adequacy criteria of a second administration within 2-14 days of the first administration [20] and an adequate agreement between the two administrations on scores for the total scale, subscales and items [18,19].

#### Face/content validity

Fifteen measures met the criteria considered adequate for face validity and content validity [33,35,37-41,43,44,48-50,52,53],[55].

#### Construct validity

Eighteen measures met the criteria for adequate construct validity [32,33,35,37,38,41-44,46-50,52-55]. Sixteen measures conducted factor analyses [32,33,35,37,41-44,46-50,52,53],[55] (although only seven reported eigenvalues) [33,41,44,47,48,50,53], nine measures examined convergent validity (r >0.40) or divergent validity (r < 0.30) with existing instruments [35,38,42,46,47,49,52-54] and six measures demonstrated significant differences on scores between known groups [35,38,41,42,53,55].

#### Cross-cultural adaptation

Three measures were re-validated with non-English speaking populations. The EORTC IN-PATSAT32 was validated with Sri Lankan cancer patients [56]; the Modified version of the Perceived Involvement in Care Scale (M-PICS) was validated with Lithuanian cancer patients [57]; and the Oncology Patients’ Perceptions of the Quality Nursing Care Scale (OPPQNCS) was validated with Turkish cancer patients [58].

Table 5 summarises which PROMs met the psychometric criteria considered adequate, as described above.

### Psychometric properties of PROMs containing all six IOM patient-centred care dimensions

The Indicators (Non-small Cell Lung Cancer) measure [40] was the only PROM that contained items covering all six IOM dimensions of patient-centred care. This measure met the criteria considered adequate for face/content validity, but not for any other psychometric criteria evaluated in this review.

## Discussion

This is the first review to identify how many of the six IOM-endorsed dimensions of patient-centred care [1] are covered in existing PROMs assessing the quality of cancer care. Our findings demonstrate that since the publication of the IOM’s *Crossing the Quality Chasm* report in 2001 [1], only one of 21 patient-centred cancer care instruments, the Indicators (Non-small Cell Lung Cancer) measure, included questions relating to the six IOM dimensions of patient-centred care [40]. However this measure only met the criteria considered acceptable for face/content validity. Further psychometric testing of the Indicators (Non-small Cell Lung Cancer) measure is required before more definitive conclusions can be drawn about its reliability and validity.

Across measures, the most commonly included patient-centred care dimensions were “information, communication and education” (19 of 21 measures) followed by “respectful to patients’ values, preferences, and expressed needs” (16 of 21 measures). In contrast, only seven measures examined patient’s perceptions of “physical comfort” and five assessed the “involvement and wellbeing of family and friends.” Possible explanations for the lesser focus on issues related to family and friends could include: 1) researchers/health professionals perceiving issues related to information and communication as the most important features of patient-centredness; 2) that the patients and survey developers involved in item selection only wished to focus on specific aspects of care; and 3) issues related to family and friends are considered a less crucial feature of cancer care. Furthermore, the measures may not have adequately captured the IOM’s six dimensions of patient-centred care because they were not developed for that purpose. For example, a measure’s objective may have been to focus solely or primarily on physical comfort, rather than to address the IOM’s six dimensions of patient-centred care. Nevertheless, the lack of PROMs that included all six IOM dimensions of patient-centred care [1] limits the potential of these existing measures to capture the *whole-person orientation* of health care and is likely to result in an incomplete representation of the quality of care provided to cancer patients.

Improvements to the reliability of existing patient-centred care PROMs and better reporting of their internal consistency, are needed. Only seven of the 21 measures met the criteria considered adequate for internal consistency by reporting a Cronbach’s alpha >0.70 for the total scale and each sub-scale [33,42,43,46-48,55]. A further six measures showed that all subscales had a Cronbach’s alpha >0.70 [34,35,44,52-54], but failed to report the internal consistency for the total scale. However, interpretation of internal consistency findings should always consider that when a subscale has a large number of items, Cronbach’s alpha can be artificially high [59,60]. Test-retest validity was very rarely considered during the development of PROMs assessing patient-centred cancer care. Although four of the five measures that examined test-retest reliability administered a second survey within 2-14 days [33,35,38,53], none of the measures demonstrated acceptable agreement between scores for the total scale, subscales and items across the two administrations [18,19]. However possible explanations for the lack of adequate test-retest reliability among PROMs assessing patient-centred cancer care may include that: 1) patients’ experiences of care, particularly for those receiving active treatment, actually changed between the initial and second administration of the measure; and 2) completing the initial measure altered patients’ expectations of patient-centred care and as a result patients rated their care differently during the second administration of the measure. Nonetheless, future research that develops PROMs of patient-centred cancer care, or validates existing measures should examine test-retest reliability, with the aim of achieving high item-to-item agreement. Item-to-item agreement is necessary [21], as high agreement between overall subscale scores can be obtained even when corresponding items within the subscale are answered differently across the two administrations.

In terms of the validity of the PROMs developed to assess patient-centred care, most of the measures met the criteria considered adequate for face/content validity (15 of 21 measures) and construct validity (18 of 21 measures). Factor analysis was the most common strategy adopted to measure construct validity (16 measures), however, few studies indicated whether eigenvalues >1 [24] were achieved [33,41,44,47,48,50,53]. Eigenvalues are used to determine the number of subscales within the measure by applying the eigenvalues >1 rule which produces psychometrically reliable and psychologically meaningful results [24]. Thus improvements to reporting whether eigenvalues were >1 appears necessary for PROMs that examine patient-centred cancer care.

The context in which these PROMs assessed patient-centred cancer care should be considered. Most measures were developed with cancer patients recruited from hospitals or treatment centres [33,34,38-44,46-50,53-55]. Only one measure was developed with patients recruited via a population-based cancer registry [32], despite benefits of such recruitment including the ability to sample a representative group of patients at different stages of the disease and with varied experiences of cancer care [61]. Although measuring the quality of patient-centred cancer care during initial treatment and hospital visits is crucial, undertaking such assessments with cancer survivors who no longer visit the hospital regularly is also important. For instance, women diagnosed with breast cancer have reported that the quality and duration of their follow-up consultations with clinicians had declined compared to the quality and duration of their initial treatment experiences [62].

The limitations of this review include that studies available in a non-English language peer-reviewed journal and the grey literature were excluded which could have led to some bias in the findings. Furthermore, the survey developers’ reasons for constructing the PROM should be considered. It is possible that the PROM’s objective may have been to focus on specific features of patient-centred care rather than to include items that covered the IOM’s six dimensions of patient-centred care. This may explain why most PROMs did not adequately address the IOM’s six dimensions of patient-centred care. Additionally, insufficient or unavailable reporting of the 21 PROMs’ psychometric properties may have influenced the ratings regarding the adequacy of the measure’s psychometric properties. We did not contact the authors of each PROM to enquire if additional unpublished psychometric information was available for that measure.

## Conclusions

Quality improvements to the health care system can be guided by PROMs assessing the quality of patient-centred cancer care. The Indicators (Non-small Cell Lung Cancer) measure [40] was the only identified PROM that included questions relating to the six IOM endorsed dimensions of patient-centred care [1], however psychometric inadequacies and/or incomplete reporting indicates that further psychometric testing of this measure is required. Using more than one measure or further developing existing measures to include all six patient-centred care dimensions could improve the assessment and the delivery of patient-centred care. Additionally, given the lack of psychometrically rigorous PROMs developed to assess patient-centred cancer care that capture the six IOM dimensions, the construction of new comprehensive measures whose psychometric properties are adequate may also be warranted.

## Abbreviations

IOM: Institute of Medicine; PROMs: Patient-reported outcome measures.

## Competing interests

The authors declare that they have no competing interests.

## Authors’ contributions

FT, SKR, RWSF, TCM, MLC and CLP were involved in study conception and design of the systematic review. FT and SKR undertook literature searches, coded the studies for eligibility and evaluated and extracted information from eligible studies. FT drafted the manuscript. All the authors revised the article critically and approved the final version of the manuscript.

## Pre-publication history

The pre-publication history for this paper can be accessed here:

http://www.biomedcentral.com/1471-2407/14/41/prepub
